# Droplet-Mediated Size Control of Stöber Silica Particles in Surfactant- and Organic Solvent-Free Emulsions

**DOI:** 10.1021/acs.langmuir.6c05490

**Published:** 2026-10-28

**Authors:** Aaron Justin Koenig, Jon Scouten, Marvin Münzberg, Ilko Bald

**Affiliations:** 426790 University of Potsdam, Institute of Chemistry, Potsdam 14469, Germany

## Abstract

The aqueous Stöber synthesis is a promising organic solvent-free variant of the conventional Stöber synthesis. However, particle formation under aqueous, phase-separated conditions remains underexplored. Here, we develop a method for tuning the particle diameter from 80 to 350 nm by varying the stirring energy introduced into the two-phase system from 70 to 1050 mW/kg. TEOS droplet sizes, ranging from 50 to 800 μm and decreasing with an increase in stirring energy, were monitored to rationalize silica particle growth rates and final particle size. Particles and TEOS droplets were characterized in line by photon density wave spectroscopy and process microscopy and off line with light scattering techniques and electron microscopy. The final particle size distributions were highly sensitive to temperature. Below 20 °C, they were aggregation-dominated, whereas an increase in temperature reduced the final particle size. The lowest polydispersity was obtained at 25 °C. The results indicate that final particle size and growth rate decrease with an increase in TEOS droplet size. Direct control of particle size, high solid content, and low organic residue content in the final product were achieved. The resulting particles exhibited 6.6 ± 1.8 nm mesopores, in contrast to conventional dense Stöber particles, which are typically nonporous or predominantly microporous. A deeper understanding of the role of process parameters in this synthesis may enable greener and more sustainable routes to Stöber silica with an increased space time yield.

## Graphical abstract

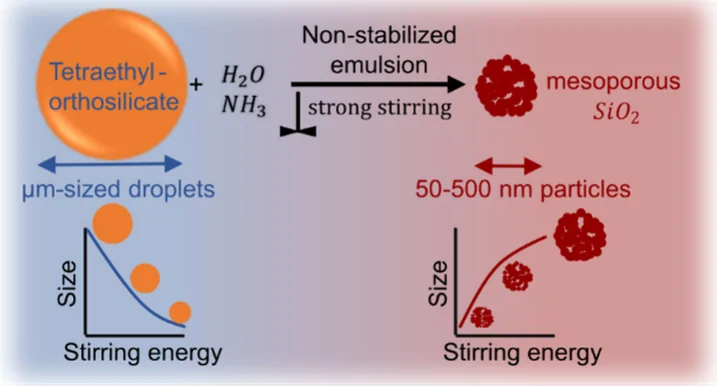

## Introduction

The synthesis of monodispersed silica particles in the submicrometer to micrometer size range is commonly known as the Stöber process and is one of the most extensively studied sol–gel processes. In the conventional procedure, water, ethanol, tetraethyl orthosilicate (TEOS), and ammonia are combined under stirring, yielding silica particles ranging from a few nanometers to several micrometers. The method was initially developed by Stöber, Fink, and Bohn^1^ and later expanded by Bogush et al., Matsoukas et al., and others.^2–6^ Early research focused on the influence of chemical parameters such as the type of silicic acid ester, solvent, reagent concentrations, and salt addition on particle growth and final particle size.^7,8^ Importantly, primary 1–10 nm particles are first generated,^9,10^ which then aggregate with each other. This aggregation of primary particles has been modeled using a Smoluchowski population-balance approach^6^ and occurs in competition with monomer addition, as described by the LaMer model.^11^ At low TEOS concentrations and slow reaction kinetics, monomer addition is thought to dominate particle growth.^9^

Stöber particles have been extensively investigated for applications in biomedical science, including drug delivery and imaging.^12^ They are also used in catalysis, ceramics, pigments, particle standards, and many other fields.^13^ A detailed understanding of the particle-formation mechanism enables the tailoring of particle properties for specific applications. However, conventional dense Stöber particles are not always desirable, because their smooth surface and typically low porosity limit their use in applications that require a large specific surface area together with small particle sizes.

A frequent challenge in Stöber synthesis is the appearance of a large miscibility gap at moderately high water concentrations or upon omission of the organic solvent; this regime has rarely been explored in detail.^14^ This lack of research is likely due to the difficulty of controlling stable sol formation in immiscible systems, where gelation or aggregation of silica particles can occur as a result of unfavorable interfacial mass-transfer kinetics. Bourebrab et al. demonstrated this limitation in systems containing ethanol as a cosolvent. At water contents above 50 wt %, the particles aggregated and their structure became undefined.^15^

To the best of our knowledge, one of the few investigations at industrially relevant concentrations within the aforementioned miscibility gap was reported by Wang et al., who recently explored the synthesis of Stöber silica particles with complete omission of the organic solvent.^16^ This resulted in a two-phase emulsion. Although their experiments were conducted well above the ammonia and water concentration ranges typically used in conventional Stöber synthesis, they successfully synthesized silica particles over a wide range of sizes by careful variation of reagent concentrations. Increasing the ammonia concentration produced particles with sizes comparable to those that can be achieved in the classical Stöber process, without agglomeration or exclusive formation of very small particles.^15,17^ Notably and of particular relevance to the present work, the experiments by Wang et al. were likely not conducted under high-shear conditions, and the TEOS droplet size was not considered as a process parameter. Furlan et al. reported a method by which <5 nm silica shells were grown on magnetite particles using only an ammonia/water solvent.^18^ They reported that shaking and sonication were required to homogenize the immiscible TEOS; however, their TEOS concentration was only 0.1 M. Deng et al. synthesized 360–770 nm silica particles by a similar method but used vinyltriethoxysilane (VTES) and homogenized the solution before the addition of the ammonia catalyst.^19^ The VTES concentration was only 0.27 M, and the particles had different characteristics due to the presence of the vinyl groups.

In this work, we aim to better understand the formation of silica particles under phase-separated conditions, particularly at high TEOS concentrations without aggregate formation. Knowledge of the processes governing the stability of syntheses carried out in purely aqueous solvents could enable more cost-effective and greener synthesis methods for both industrial application and routine research use.

Process parameters are known to play an important role in the conventional Stöber synthesis, and their influence on the aqueous Stöber synthesis is explored here. We initially hypothesized that reagent homogeneity within the reaction vessel affects particle growth rates and final particle size, specifically because the reaction is of a pseudo-second-order nature and no depletion layer of reagents is formed.^20^ The stirring energy, stirrer type, reactor geometry, and reaction temperature were found to strongly modulate silica particle growth rates and final size. In the reactor geometry employed here, TEOS droplets are generated by turbulence and shear forces included by the stirrer. These droplets were measured by in-line microscopy, showing that an increased stirring energy produces smaller TEOS droplets. The influence of TEOS droplet size on final particle size and growth rate is then discussed. The process was additionally monitored in line with photon density wave spectroscopy (PDWS), which independently measures the absorption and scattering in highly turbid dispersions.^21^ PDWS was used to identify the end of the reaction, defined by the absence of further changes in particle number or size.

Several advantages of this method over the conventional Stöber reaction in an organic solvent are demonstrated. First, the synthesis time is reduced significantly, with growth stopping after at most 1 h. Second, a high mass fraction of 8.7% is achieved without seeded growth.^5^ Third, the stirring energy is introduced as a process parameter for controlling final particle size. Reagent concentrations are fixed in this work in order to isolate only the effects of mixing, reactor geometry, and temperature. Finally, we show that the produced particles are mesoporous, which we attribute to aggregation-denominated growth and a low degree of condensation.

## Results and Discussion

The reaction scheme is briefly described in Figure 1 and is approximated well by a flat interface between the TEOS and aqueous ammonia phase.^20^ Water, ammonia, and TEOS are combined under turbulent stirring, producing an unstable emulsion of TEOS droplets in the aqueous phase. Uniform, spherical, and well-dispersed silica particles are obtained in a short time compared to regular Stöber silica. An example of a dispersion obtained by this method is shown in Figure 1b. The influence of temperature, stirring energy, and TEOS droplet size on the growth and final particle size is discussed herein.

**Figure 1:**
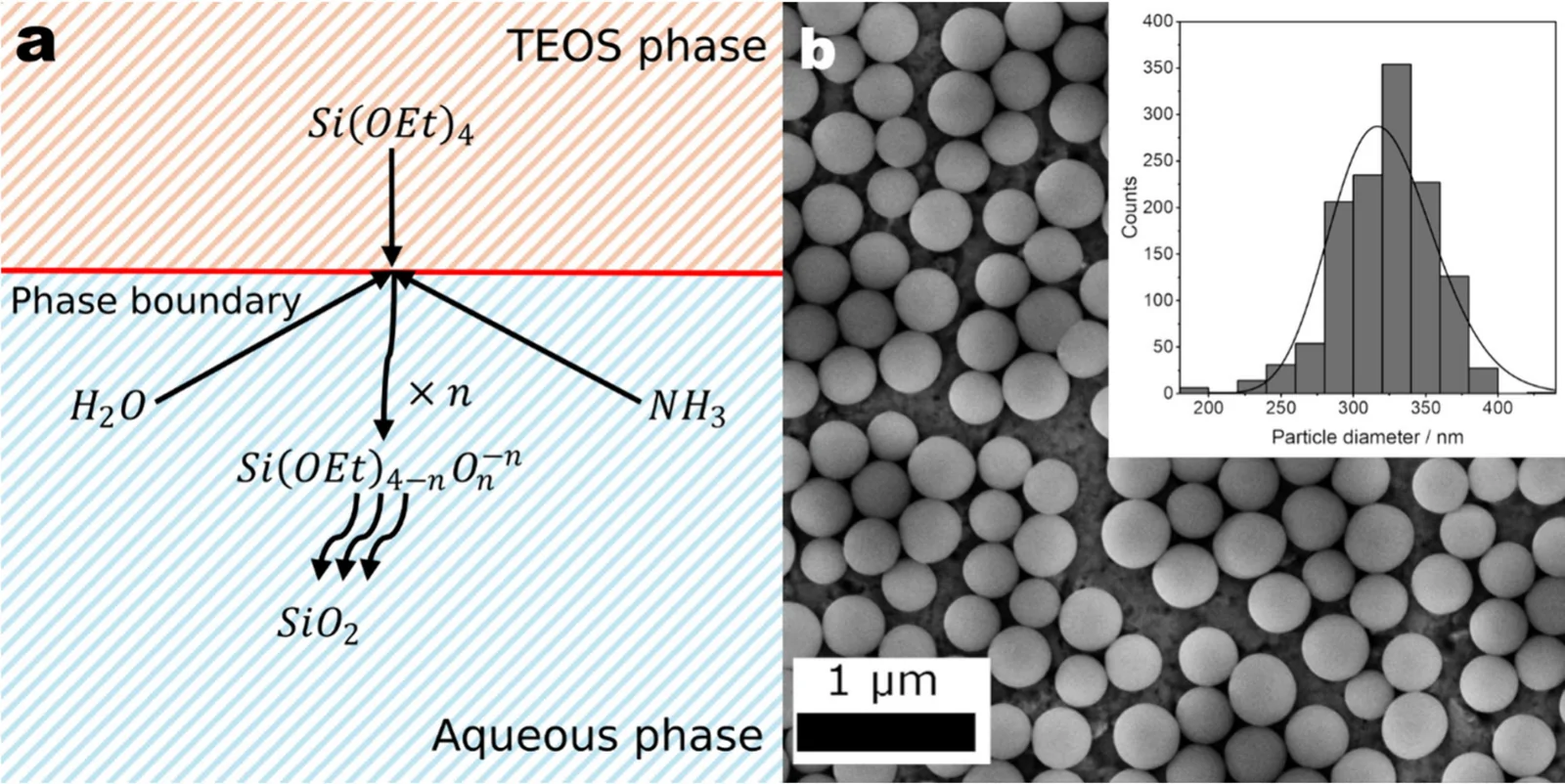
(a) Schematic overview of the reaction scheme in the organic solvent-free modified Stöber synthesis. Droplets of TEOS (orange area) are generated due to turbulence from the stirrer and reactor geometry. The hydrolysis reaction of TEOS occurs at the interface layer between TEOS and the aqueous ammonia phase (red line), after which generated orthosilicic acid condenses to form the well-known Stöber silica particles in the aqueous phase (blue). The interface is approximately flat due to the large radius of curvature. (b) Scanning electron micrograph of the resulting silica dispersion with its distribution and log-normal fit thereof shown in the inset. The particle size distribution was measured over at least 500 particles from different images, which are not shown here.

We observed that the stability of the system is sensitive to temperature. Below about 25 °C, particle aggregation dominates and smaller particle populations disappear over time. Above this threshold, silica particles (SPs) with a narrow size distribution are obtained. The final SP size was previously reported to be inversely proportional to temperature, and our results corroborate this observation.^22^ The effect of temperature is discussed in depth below. We observed that final SP size is proportional to stirring energy, while the polydispersity index (PDI) remained constant. The PDI is comparable to that of traditional Stöber syntheses containing ethanol, which is remarkable considering the phase-separated nature and nonuniform size of TEOS droplets in the dispersion. Smaller droplets of TEOS were observed under higher stirring energy input. The droplet size is reduced over time due to droplet breakage, TEOS hydrolysis, and stabilization due to the Pickering effect.^23^ First, the resulting dispersions are analyzed, and then effects of temperature on particle growth are demonstrated. To gain a deeper understanding of the mechanism driving changes in particle size with a variation in stirring energy, the temporal evolution of TEOS droplet size in relation to stirring energy is examined. The reaction kinetics are briefly investigated to explain the effect of TEOS droplet size on final SP size.

### Particle Morphology

Particles synthesized by the organic solvent-free method maintain a spherical geometry, just as with the classical Stöber synthesis.^16^ Deviations occur when particles are small, likely caused by surface roughness due to the size of primary particles.^9,24,25^ The difference in particle morphology resulting from both methods is visually compared with scanning electron microscopy (SEM), showing that similar morphologies are obtained from the classical Stöber synthesis (Figure 2a) and organic solvent-free SP synthesis (Figure 2b). In Figure 2c, at a low stirring energy, a large proportion of the observed particles appear fused, a result of aggregation of secondary particles that do not further aggregate but continue to grow. A similar effect of secondary–secondary particle aggregation was observed by Wang et al. when small amounts of water or ammonia are present (<30 or <8 M, respectively), although only the largest particles were affected, not the smallest as observed here.^16^ Bogush et al. noted that for Stöber syntheses, the aggregation of secondary particles with each other may be neglected.^6^ This does not appear to be applicable for the organic solvent-free synthesis in cases in which the stirring energy or temperature is low. Nevertheless, particle morphology is well preserved at a high stirring energy, while particle aggregation dominates at a lower stirring energy and small particle sizes. In addition, sphericity is maintained just as with classical Stöber syntheses, with no major differences observed between the two methods.

**Figure 2:**
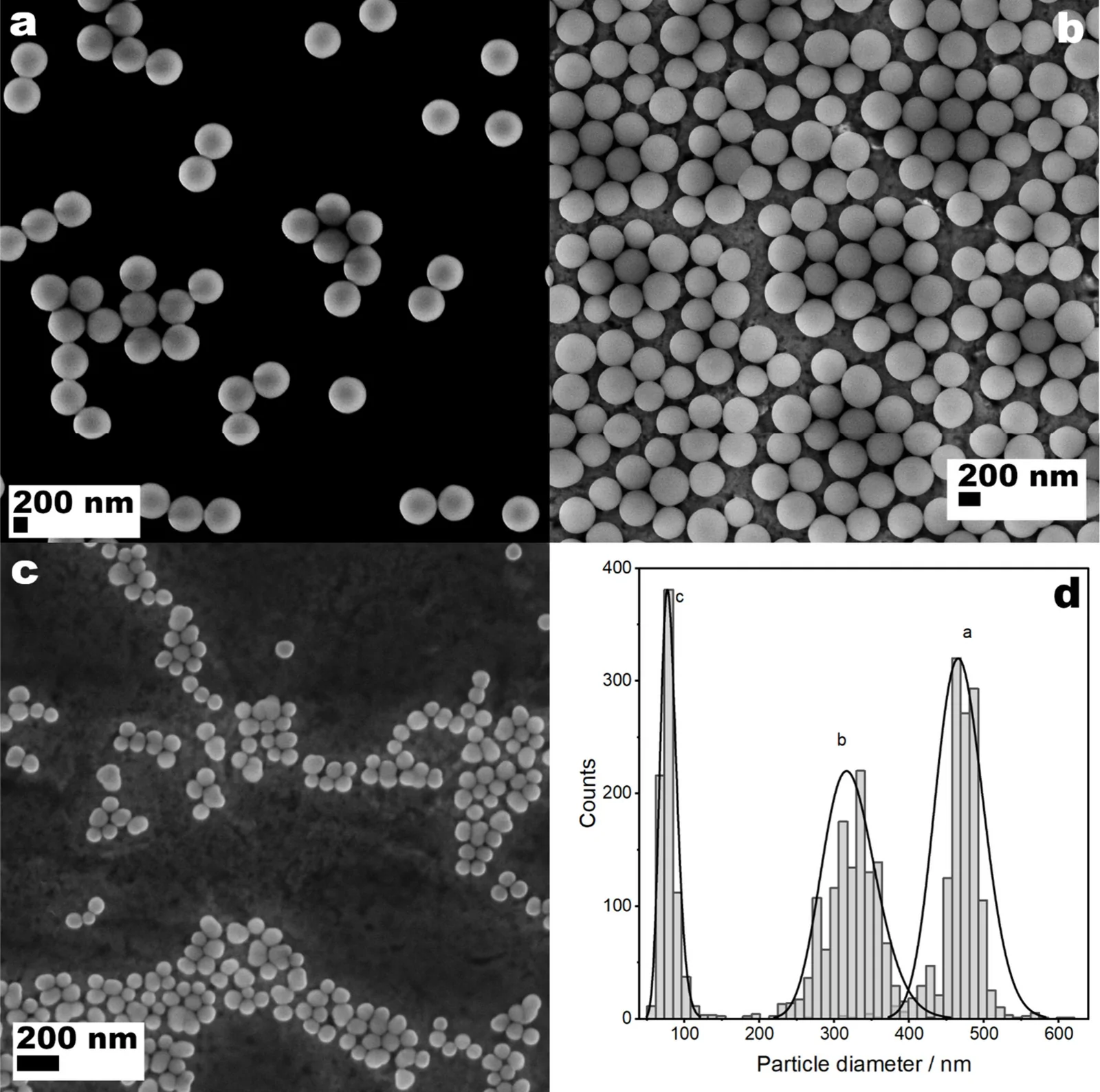
(a) Silica synthesized by the classical Stöber method. (b) Silica synthesized by the ethanol-free Stöber method at a stirring energy of 1.08 W/kg. (c) Silica synthesized by the ethanol-free Stöber method at a stirring energy of 8 mW/kg. (d) Particle size distributions of the three syntheses described above with log-normal fits. At least 500 particles were evaluated.

### Effect of Temperature on Particle Growth and Particle Size

Changes to the reaction temperature were observed to have a significant effect on the aggregation and particle size of SPs (Figure 3a). The reaction temperatures at 25 °C resulted in the narrowest distributions, likely close to the optimal reaction temperature at the investigated reagent ratios. The data shown in Figure 3a demonstrate that the number and size of formed aggregates increased with a decrease in temperature. At 20 °C, the submicrometer particle population persists but is accompanied by a substantial fraction of aggregates. Below 20 °C, the submicrometer particle populations disappear due to aggregation. Nevertheless, the final SP size is inversely proportional to reaction temperature. This is in agreement with previous publications.^26^ The number of aggregates formed at or above 25 °C is low for Stöber syntheses in the miscibility gap; it is typically expected that only very small particles (<50 nm) and large aggregates form at these high concentrations of water.^15,16^

**Figure 3:**
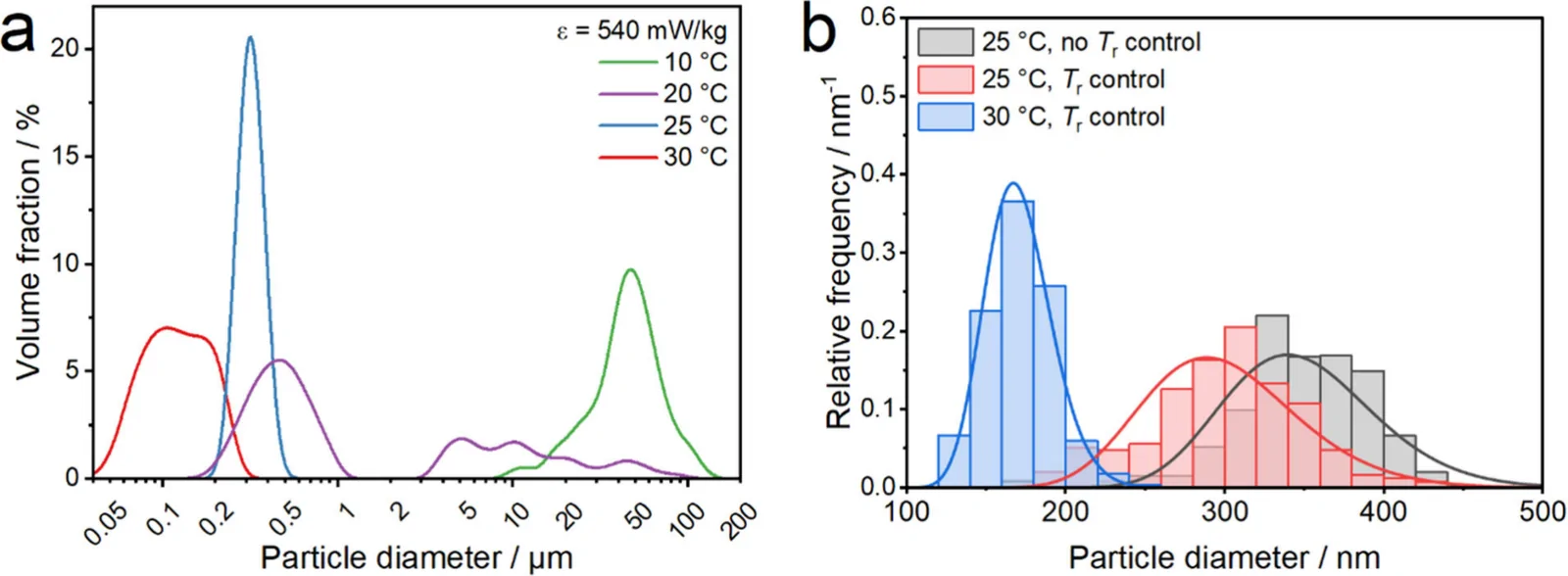
(a) Static light scattering volume distributions for syntheses with mixtures stirred at 400 rpm (540 mW/kg) at different set temperatures. Distribution widths at sizes of <0.2 μm are inaccurate and should not be considered. (b) Comparison of SEM particle size distributions obtained at stirring energies of 540 mW/kg without control of reactor temperature *T*_r_ (black), with control of *T*_r_ at 25 °C (red), and with control of *T*_r_ at 30 °C (blue). Lines indicate log-normal fits to the distributions.

The classical Stöber process starts with an induction period during which the TEOS monomer is hydrolyzed, but no particles are formed.^27^ This was observed by measuring the reduced scattering coefficient (μ_s_′) with photon density wave spectroscopy (PDWS), which changes based on the number and size of particles in the dispersion (Figure 4). The reaction mixtures rapidly became opalescent after approximately 3 min. For all experiments, few but large agglomerates 3–100 μm in size are observed in process microscopy images. These agglomerates occur regardless of the stirring energy or other process conditions but comprise a small fraction of the final mass of particles for syntheses at ≥25 °C, typically less than 5 wt % of the total mass of silica (see Figure S4a).

**Figure 4:**
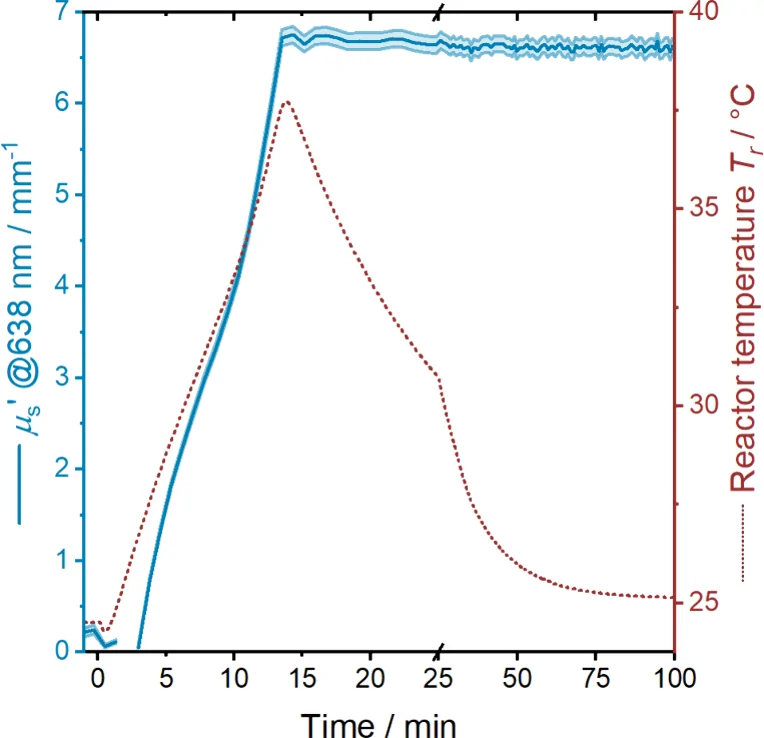
Reactor temperature for experiment 13 (see Table S1, which provides an overview of experiments) stirred at 400 rpm (540 mW/kg). Reactor temperature *T*_r_ (red) was not controlled by closed loop PID control. The reduced scattering coefficient is recorded by PDW spectroscopy (blue). Note the change in the time scale after 25 min.

Monodisperse dispersions were obtained for temperatures of 25 and 30 °C (see Figure S4b,c). In previous works, monodisperse dispersions were obtained up to a minimum of 9 °C for ethanolic Stöber syntheses,^5^ likely a similar effect to the aggregation observed herein, although examples of syntheses at temperatures as low as −20 °C have been published.^22^ The reason that aggregation occurs is unclear from these data, likely caused by several effects occurring simultaneously. These may include changes in the viscosity and Derjaguin–Landau–Verwey–Overbeek (DLVO) energy barriers of the dispersion, resulting in shear-induced aggregation, which is especially prominent for larger particles and may occur rapidly if the shear rate is high and the DLVO potential barrier is low.^28,29^ We tentatively attribute a large portion of the aggregation to reactive hydrolyzed TEOS species with low charge, which are not repelled and may cross-link growing particles into aggregated networks. Utilizing the framework presented in the literature, a decrease in aggregation time with a higher viscosity and larger nanoparticle size is expected. The complexity of the system made it difficult to isolate these variables, and further experimental evidence is required.

When the reactor temperature was not controlled, the reaction temperature increased from 25 to ∼38 °C after 14 min (Figure 4). μ_s_^′^ reaches a steady state at the same time as the reaction temperature. This is an indication that the hydrolysis is slower than particle growth, as otherwise μ_s_^′^ would continue to increase after the reactor temperature started to fall. Monodispersity was maintained, and the SPs grew to a size larger than that of particles synthesized at a constant temperature of 25 °C (Figure 3b), contrary to the literature that holds that higher temperatures always resulted in smaller particle sizes. The influence of temperature before the induction period is as expected; lower temperatures result in larger SPs.^22^ The decreased overall viscosity at increased temperatures allowed the production smaller droplets (Figure 5), while the induction phase of the reaction is unaffected due to the delayed change in the reactor temperature.^30^

**Figure 5:**
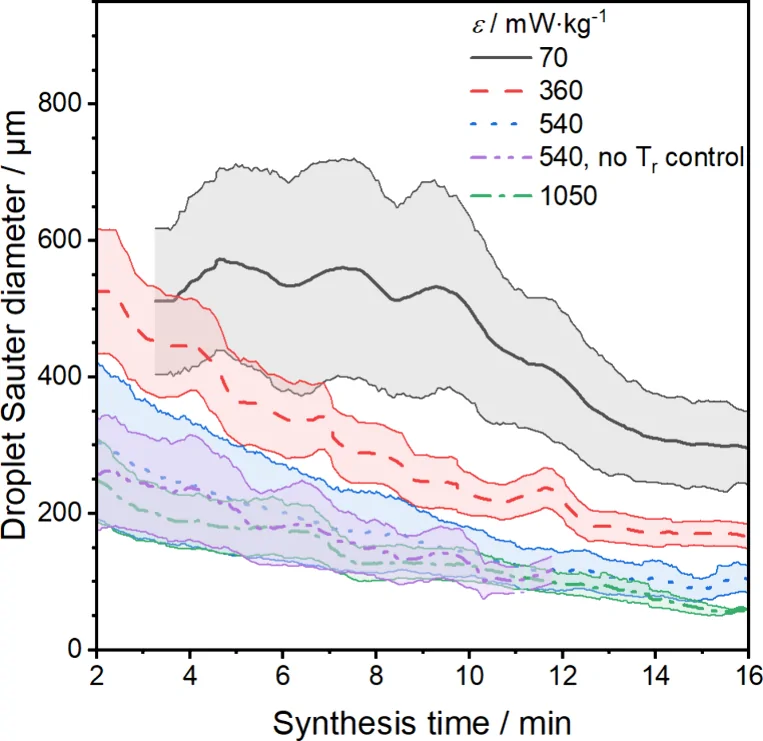
Sauter mean droplet diameter measured by in-line microscopy for five syntheses at different stirring energies *ε*. One of the syntheses was performed under nonisothermal conditions, and the temperature was allowed to increase from 25 to 37 °C. Micrographs were evaluated every 2 s, but data here are smoothed in a 1 min window. Semitransparent areas denote the standard deviation in size. Note that the process microscope is limited to <1 μm diameter droplets.

In order to compare the obtained reaction profiles to the literature, the enthalpies for complete hydrolysis and condensation were estimated by integration of the dotted curve in Figure 4 to the end of the reaction. A value of −32 ± 5 kJ/mol was obtained. This value falls between the values calculated by Matsuoka et al., Wood et al., and Tiller et al. (−12.9, −38, and −43.9 kJ/mol, respectively).^31–33^ This uncertainty should be resolved in future works for better characterization of the system. Twenty-five degrees Celsius was chosen as the preferred temperature for further experiments due to narrow distributions and minimal aggregation of SPs. For scale-up, care must be taken to avoid thermal runaway due to the high concentration of TEOS (30 wt %). This has hitherto not been an important issue with Stöber syntheses due to much more dilute TEOS concentration, typically less than 10 wt %.

### Impact of the Stirring Energy on TEOS Droplet Size

Due to the miscibility gap in the TEOS/water/ammonia system, two phases are obtained, which react only at the interface. If nonturbulent stirring were utilized (as in a beaker), the phase boundary would be clearly maintained and reaction rates would be low due to the minimal interface area. Herein, the two-phase system is emulsified by utilizing a large stirring implement with additional baffles in a 1 L round-bottom cylindrical reactor with rapid stirring (see Experimental Section). Droplets of TEOS are generated due to stirrer turbulence, their size ultimately depending on the stirring energy input of the stirrer into the system.^34,35^ Stirring energy (or power consumption) *P* for complete suspension was calculated by eq 1.^36^

1 $$P=N_{p}N^{3}D^{5}\rho_{c}$$

where *N*_p_ is the stirrer and reactor geometry specific power number, here assumed to be 0.34,^37^ *N* the stirring rate, *D* the diameter of the stirrer (0.08 m), and ρ_c_ the mean density of the dispersion, here 977 kg/m^3^ at the beginning of the synthesis. The quotient of *P* with the total reaction mass yields specific stirring energy *ε*. Reynolds number *Re*, as an indicator for turbulent mixing, was calculated by eq 2.^38^

2 Re=ρcND2μ

where μ is the bulk viscosity of the fluid. The viscosity of TEOS (0.6 mPa s) and water (1 mPa s) may appear to be good assumptions at first. However, bulk viscosities in emulsions are often much higher than in the individual phases, depending on many factors such as the temperature, volume fraction of the dispersed phase(s), and droplet size distribution (DSD).^39,40^ The problem is complex, with viscosity in a likely range of 2–10 mPa s, depending on shear rate.^41^ Ultimately, 2 mPa s was chosen as an approximation and is used in the calculations below based on the work of Pal.^41^ The minimal value was chosen only as an approximation, as it best fit the trend of turbulent mixing at different stirring rates. An *Re* value of >10^4^ is considered turbulent flow for most reactors.^42^ For the high end of the range of viscosity at 10 mPa s, the Reynolds numbers would be significantly lower, but no phase separation was observed above 100 rpm; thus, this value is discarded. The results of the calculations are listed in Table 1.

**Table 1:** Theoretical Reynolds Numbers, Stirring Energies, and Specific Stirring Energies in Relation to the Stirring Rate

*N* (min ^–1^)	100	200	300	350	400	500	1000^a^
*Re* (×10^4^)	0.5	1	1.6	1.8	2.1	2.6	0.7
*P* (mW)	5	40	140	220	320	630	0.21
*ε* (mW·kg^–1^)	8	70	230	360	540	1050	1191

a The rotor-stator mixing geometry was determined with a Silverson L5M-A instrument. The total reactant mass was 0.185 kg instead of 0.6 kg for the other experiments.

It is expected that turbulent mixing is achieved at or above 200 rpm in this reactor configuration. Uncertainty in *Re* and *ε* arises due to the reactor and stirrer geometry, temperature, volume fraction of TEOS, side products such as ethanol, and Pickering effect of the emerging particles.^43^

The turbulent mixing discussed above, along with the shearing force generated by the stirrer, causes the generation of small droplets of the TEOS phase. These droplets slowly coalesce back into their continuous phase; a balance between droplet generation and coalescence is achieved. When droplet sizes are smaller than about 1 mm, coalescence is additionally inhibited due to surface charging, especially at the high pH investigated here.^44^ For long stirring time scales, a constant droplet size is typically obtained when the medium is not reactive.^34,45,46^ Here, however, there is only a short induction period of a few minutes before the first silica particles are formed, and total reaction times are typically less than 30 min. Thus, droplets are larger than their equilibrium size. Hydrolysis of the TEOS monomer results in ethanol production, dynamically varying viscosity and miscibility. As a result, prediction of the exact DSD is difficult. Instead, droplet sizes were evaluated with the aid of a process microscope. The area evaluated was always 1 mm^2^; typically up to 10 droplets were evaluated per image, with one image evaluated every 4 s. This is not a particularly strong statistic but gives a reasonable estimate of average droplet size and distribution width. In the induction period of the reaction, droplet size evaluation is difficult due to their large size (i.e., larger than the field of view) and low contrast, as the in-line microscope relies on backscattered light. Later in the reaction (i.e., beyond about 14 min), droplet sizes were typically small and thus difficult to resolve and few in number. The statistics here are not quantitative due to this inaccessibility. The evolution of the Sauter mean diameter with various *ε* values is displayed in Figure 5. As no emulsifiers are present, the droplets are 50–800 μm in size. The results from the manual droplet size evaluation were averaged with a moving window of 60 s.

Importantly, miscibility is achieved near the end of the synthesis because of the production of ethanol during the synthesis. This results in the disappearance of droplets from the in-line microscopy images. Miscibility is achieved when more than 95% of TEOS has been consumed, based on the ternary phase diagram of TEOS-water/ammonia-ethanol.^47^ Although miscibility is not achieved until near the end of the synthesis, dispersant characteristics do change with an increase in ethanol concentration.^47^ Wang et al. showed that when 10% of the typical amount of ethanol is used, particles grow larger than without it. It is likely that the ethanol produced during the reaction has some influence on the particle size.^16^

The total available surface area in the reacting volume, relevant for the reaction rate, is proportional to the DSD and the number of droplets, which is proportional to the ratio of the total volume to the volumetric DSD. This interface area may be calculated when the amounts of TEOS (*n*_TEOS_) and the DSD are known. Unfortunately, *n*_TEOS_ is not easily accessible but may be estimated from thermodynamic data (see below). The DSD may be approximated from data in Figure 5. Phase separation occurs in *Re* < 10^4^ systems. Therefore, the total interface area between the ammonia–water and TEOS is smaller in syntheses with slow stirring, as only a few droplets are formed and most of the TEOS accumulates in the bulk. The effect of this phase separation on the produced particles is discussed below.

### Effect of TEOS Droplet Size on Particle Growth and Final Size

It was observed that the final SP size increases inversely proportional to the TEOS droplet size. As mentioned in the previous section, the droplet size strongly depends on the mixing conditions of the reactor but especially specific stirring energy ε. Based on the data obtained, the final particle size depends on the specific stirring energy according to a logarithmic scaling law and behaves as *d̅*_particle_ = (110 ± 6 nm) × log ε + (316 ± 11 nm) in the range from 0.01 to 1.05 W/kg. The parameters of the equation strongly depend on the reagent concentrations and reactor geometry and are not meant to be general in scope. As described by Wang et al., reducing the TEOS or ammonia concentration decreases the final particle size, while reducing the water concentration increases the particle size. This is to be expected, given that particles grow only as long as there is TEOS available and the ammonia concentration is the limiting reagent.^16^

At a high ε, a plateau in SP size is evident and the TEOS droplet size decreases in proportion to the logarithmic increase in ε (see Figure 6). Decreasing the droplet size further is likely to result in still larger SP sizes. Meanwhile, the PDI of silica remains approximately constant (Figure 6) regardless of the stirring conditions, even in syntheses with phase separation. The stability ratio, determining aggregation dynamics between two particles of arbitrary size, thus does not vary significantly with ε.^6^ As previously stated, for the experiment with no temperature control, the droplets were overall slightly smaller. A much shorter reaction time was observed (14 min vs 20 min). This could explain the discrepancy in the final particle size observed (Figure 3b). While the stirring conditions in the rotor-stator mixer deviate due to the use of a different reactor and stirrer geometry, smaller droplet sizes are likely produced nevertheless and result in larger particle sizes due to the higher shear rates achieved. However, complete aggregation was observed when stirring was continued for more than 10 min in this reactor geometry. No significant aggregation was observed in the syntheses in the default reactor configuration regardless of stirring duration. This is once again attributed to shear-induced aggregation, since shear rates in rotor-stator mixing geometries are approximately 10^3^ higher, in this specific example, although not experimentally confirmed.^28,29,48^

**Figure 6:**
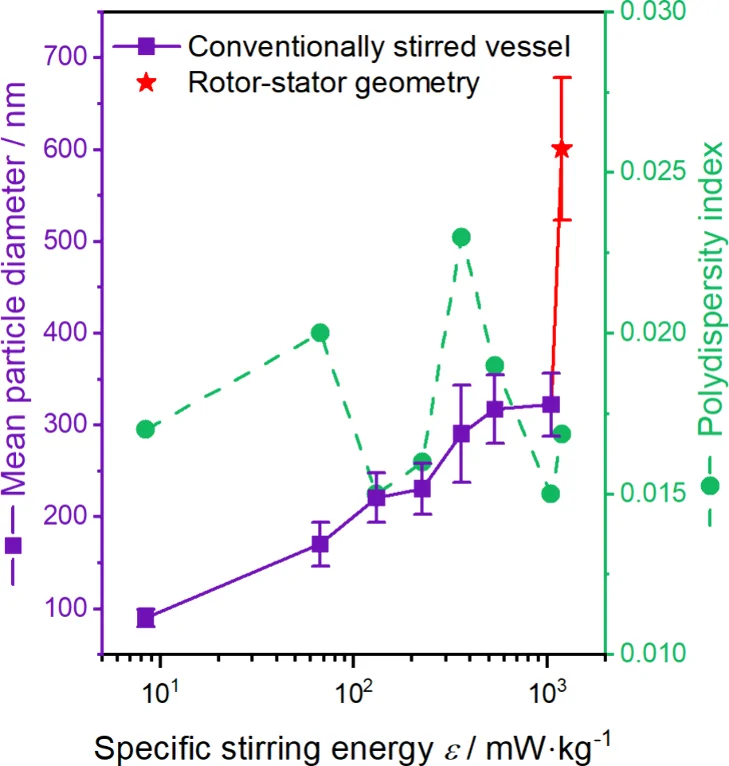
Changes in mean particle diameter and polydispersity index over a range of different stirring energies. Particle sizes and distributions are measured from SEM images. The absolute distribution width in nanometers is given by the error bars.

Because droplet size decreases at a higher ε, the overall hydrolysis rate of the TEOS precursor increases, as it is proportional to the total interface area. Evidence of this trend is presented in Figure 7, where heat evolution ceases earlier at a higher ε, consistent with the smaller Sauter diameters observed under these conditions. A similar trend is observed with PDW spectroscopy in Figure 8, where μ_s_^′^ remains constant when Δ*T* reaches its second maximum. In the phase-separated synthesis conducted at ε = 8 mW/kg, the heating period subsides significantly more slowly than in syntheses performed at higher stirring energies. This behavior indicates poor mixing of reagents and results in a substantially smaller surface area of the TEOS–water interface, concomitant with the insufficient mixing predicted by the Reynolds number.

**Figure 7:**
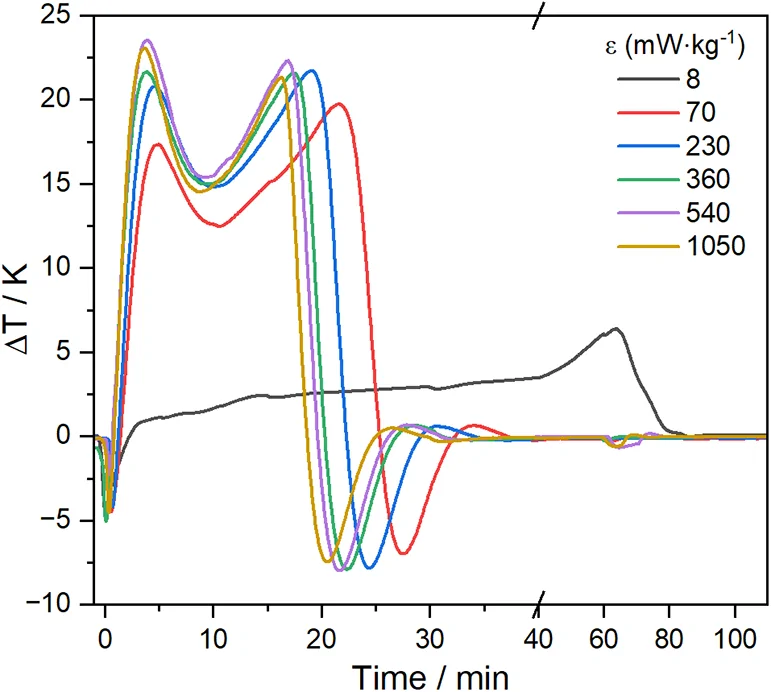
Difference in temperature between dispersion and reactor jacket, Δ*T*, investigated for different stirring energies at otherwise equal reactant ratios at 25 °C. Poor mixing at 8 mW/kg (gray line) is reflected in inconsistent heating and cooling of the reactor. The actual temperature in the reactor does not deviate more than 2 K from the set point.

**Figure 8:**
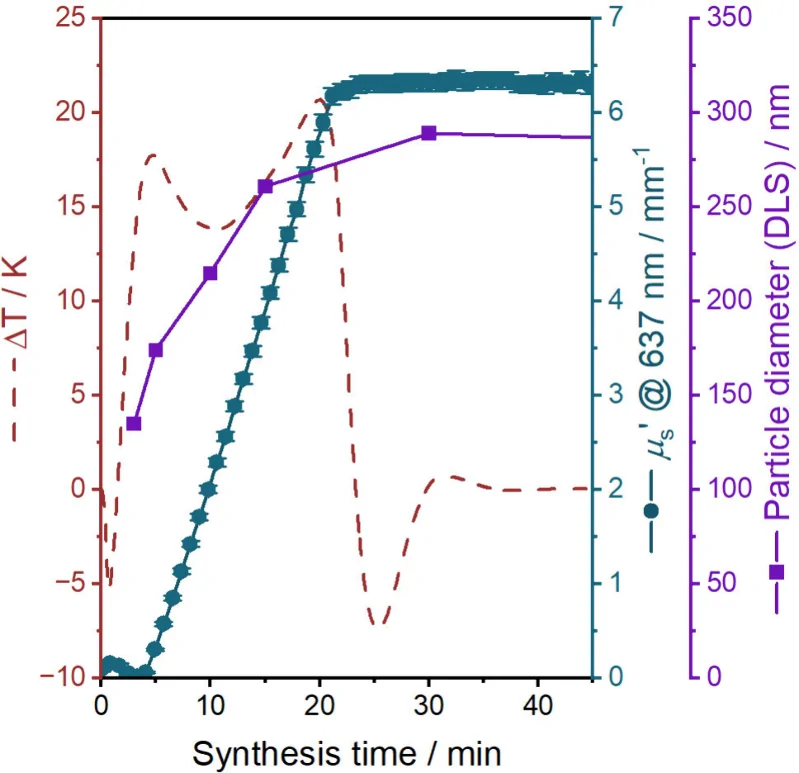
Trend for a synthesis with a specific stirring energy of 230 mW/kg. The reduced scattering coefficient (blue circles) and its error (blue shaded area), dynamic light scattering particle diameter (purple squares), and temperature difference between the reactor and the reactor jacket (red line) are plotted. Heat is released until the second maximum in Δ*T* at approximately 22 min, after which the reduced scattering coefficient rapidly reaches an equilibrium value.

If the ratio of primary particle aggregation and nucleation rate remained constant, no changes in SP size would be evident for changes in ε.^6^ This clearly is not the case; thus, the larger SP sizes are tentatively attributed to a change in this ratio. This effect cannot be explained by an increase in ε alone, as confirmed by previous publications.^49^ Simulation is difficult, and no model exists describing generation and breakage of TEOS droplets, reaction at the interface, and subsequent aggregative growth simultaneously. All three models exist separately but require a unified description.^6,20,50^

### Stirring Energy Effects on Thermodynamic and Kinetic Observations

Next, Δ*T* trends observed in these syntheses are discussed. It is to note that the absolute temperature deviates by only a small amount (typically <2 K), and the trends represent the difference in temperature between the reactor and jacket. Positive values indicate that the reactor is warmer than the jacket. Immediately upon mixing, a mismatch in temperature between reagents results in brief reactor heating due to the proportional-integral-derivative (PID) control loop.

In Figure 7, two main phases are visible for each synthesis. The first phase occurs immediately after mixing, where the hydrolysis rate reaches its maximum. The point in time for this maximum is inversely proportional to ε. A second phase appears over a larger range of time scales after the first maximum in Δ*T*. Noteworthy is the change in time scale depending on ε, suggesting a different mechanism of the second exotherm than for the first maximum. What appears to be an endothermic feature starting at around 20 min is additionally visible, signifying the end of the reaction.

After the second reaction phase, no more heat evolves and Δ*T* reaches a minimum, with some overcorrections due to PID control. The DLS and PDW spectroscopy results shown in Figure 8 indicate that particle growth quickly subsides after all reaction enthalpy has evolved. The observed negative slope in Δ*T* is thus attributed to reactor cooling rather than an endothermic process. These results suggest that the growth and aggregation rates of particles are fast compared to hydrolysis of the TEOS monomer; the concentration of hydrolysis products [Si­(OEt)_4–*n*_O_*n*_^*n*–^] (where *n* is the degree of hydrolysis of the monomer) is assumed to be low for all points in the synthesis beyond the induction period due to the faster reaction kinetics of condensation compared to hydrolysis.^27^

For nearly all syntheses carried out in this work, no change in SP size is observed beyond 45 min, with shorter reaction times observed for higher stirring energies. An exception is the synthesis stirred at 100 rpm (8 mW/kg) where phase separation occurred, and the reactor temperature did not stabilize for more than an hour (Figure 7). The reduced scattering coefficient reached a maximum at 82 min for that synthesis, coinciding with the end of the change in Δ*T*. In general, kinetics are fast compared to classical Stöber syntheses carried out in the same reactor where, regardless of particle size, at least 3 h was required until no more changes in SP size could be detected (see Figure S5).

With a higher degree of hydrolysis of Si­(OEt)_4_ to Si­(OEt)_4–*n*_O_*n*_^*n*–^, hydrophilicity increases and the particles are most probably exclusively formed in the aqueous phase. Due to the large excess of TEOS at the interface, the reaction rate is approximated by a pseudo-second-order reaction.^11,51^ To model the consumption of TEOS from the droplets, the changes in droplet size and number are significant. A Smoluchowski population balance may be employed containing a source term (droplet breakage by the stirrer) and two sink terms (droplet coalescence and hydrolysis of TEOS). Locally, the hydrolysis rate is constant regardless of droplet size due to the assumption of a planar reaction interface. The interfacial reaction rate is further explored by O’Shaughnessy.^20,52^ In essence, the reaction rate depends on the total interface area and the diffusion of reagents (in this case, only ammonia and water). Currently, kinetic evaluation is not trivial due to the presence of many factors such as a DSD with droplet generation and coalescence, the presence of particles at the interface, and variable parameters such as heat capacity, viscosity, and TEOS phase volume fraction.

It was discussed above that lower temperatures lead to lower hydrolysis rates and as a consequence larger SPs, consistent with previous studies.^1,5,22^ However, contrary to intuition, particles grew larger when the overall reaction rate was increased through a higher ε. Lee et al. argued that nucleation and primary particle aggregation rates are in competition with each other, and increasing water and ammonia concentration increases the ratio of aggregation to nucleation rate.^53^ As a consequence, fewer particles that grow emerge. In the case discussed herein, the rate of aggregation or nucleation is likely limited, and their ratio changes at more rapid hydrolysis rates. This stands in contrast to a homogeneous system in which the TEOS, ammonia, and water concentration and their diffusion rate modulate the ratio.

### Elemental and Pore Size Analysis

The elemental composition of the herein synthesized particles was examined using a wet chemical method. The results are listed in Table 2 and strongly suggest that less ethanol is incorporated into the particles synthesized by the ethanol-free method as opposed to the Stöber method. This could positively impact the performance and toxicity of Stöber silica or enable the use of particles without prior calcination.^54^ The poor solubility of TEOS in the aqueous phase likely favors a more complete hydrolysis, resulting in fewer ethoxy moieties (C_2_H_5_O). Due to the low final ethanol concentration and high water content during synthesis, re-esterification of free silanols is likely reduced.^55^ Although previous work suggests an incorporation of solvent into the particles, less water is present in the SPs synthesized herein.^5^ This is likely a side effect due to the increased porosity as evidenced by transmission electron micrographs (Figure 2d), reducing the frequency of blind pores containing solvent. The composition of the investigated SPs was calculated from Table 2a. All silicon was assumed to be in the form of SiO_2_ and all carbon present due to ethoxy moieties. The remaining hydrogen is assumed to be due to the presence of water. The results of this calculation are listed in Table 2.

**Table 2:** (a) Elemental Analysis of Two Silica Samples, One Synthesized by the Classic Stöber Method and the Other by the Organic Solvent-Free Stöber Method,^a^ and (b) Calculated Molecular Compositions from Part (a)

Mass percentage of the element or compound	Stöber synthesis	Organic solvent-free Stöber synthesis
(a) Elemental Masses		
wt % C	1.93	0.10
	1.94	0.11
wt % H	1.62	0.90
	1.63	0.91
wt % Si	40.42	42.94
	40.40	42.93
wt % O (calculated)	56.0	56.02
	56.1	56.02
wt % N	<0.01	<0.01
	<0.01	<0.01
(b) Resulting averaged molecular formulas		
wt % SiO_2_	86.45	91.85
wt % H_2_O	9.8	7.88
wt % C_2_H_5_O	3.63	0.21
wt % NH_3_	<0.01	<0.01
wt % unaccounted	0.11	0.05

a Measured in duplicate. The weight percent denotes the percentage of mass of the total sample mass.

The results for the ethanol-free silica are comparable to the findings of Bogush et al. and Van Helden et al.; the exact drying method of the SPs likely has some influence on the obtained results. The carbon (or ethoxy group) content for the classical Stöber synthesis in this work is slightly higher than those found by other groups (0.15% and 0.8%, respectively), likely due to variations in the reagent concentration as well as process variation, since van Helden et al. obtained a value of 0.8% even after drying at 180 °C in vacuum.^5,56^ The nitrogen content of both samples was below the limit of detection, suggesting ammonia is fully desorbed upon drying.

Brunauer–Emmett–Teller (BET) analysis was performed for one representative sample (see entry 5 of Table S1 and adsorption isotherm S7). The measured specific surface area (SSA) was small (22.3 m^2^/g), likely influenced by sample preparation and storage conditions.^57^ While only SSA values are often reported, the pore size distribution is equally critical for performance in applications yet remains underreported despite its strong impact on mass transport, accessibility of active sites, and loading of active species. Pore size distributions derived using the Barrett–Joyner–Halenda (BJH) model showed an apparent maximum at approximately 60 nm, a value that is inconsistent with observations from HRTEM (see section S6). When the specific surface area of the sample is considered, it is obvious that the measured value by N_2_ adsorption is largely represented by the geometric surface area of smooth, nonporous nanoparticles that were 127 nm diameter.^58^ Determining the pore size distribution from HRTEM (Figure 9) yielded a more physically meaningful distribution (Figure S6) with a maximum of around 5 nm. This value is slightly larger than previously published data, where pore diameters between 1 and 3 nm are typical.^57,59^ Sample preparation for nitrogen adsorption measurements is nontrivial, and the method utilized herein likely closed most of the pores. Shortcomings of the method are critically discussed elsewhere.^60^ Nevertheless, the porosity observed here is markedly different from that of regular Stöber silica, where mostly micropores are observed instead. Importantly, the observed mesoporosity is achieved without the use of organic templates, avoiding additional template-removal steps and associated processing complexity. The particles are preserved in their native state without shrinkage or changes in the gel network due to sintering.^61^ Filipović et al. synthesized mesoporous silica with pore sizes centered around 4.5 nm with a high molar ratio of water to TEOS as well as a relatively high ammonia concentration.^62^ They argue that high a TEOS concentration in the reacting phase causes the formation of smaller pores. In contrast to a traditional Stöber synthesis, the aqueous phase here contains no TEOS, but only its hydrolysis products, which accounts for the preservation of open pores in the present syntheses. In addition, they noticed that aging of the sol closed off pores. Chen et al. further observed that the degree of condensation of the silica network at such high ratios of water to TEOS is low, and washing with water in the presence of residual ammonia effectively removed poorly condensed silica structures.^59^ This led to the formation of mesoporous silica. The experiments here bear striking similarity, differing in the complete initial absence of an alcoholic solvent. We tentatively attribute the observed porosity to the incomplete condensation of the silica network and postsynthesis washing with water aids in revealing the mesoporous structure, but further experimental evidence is required.

**Figure 9:**
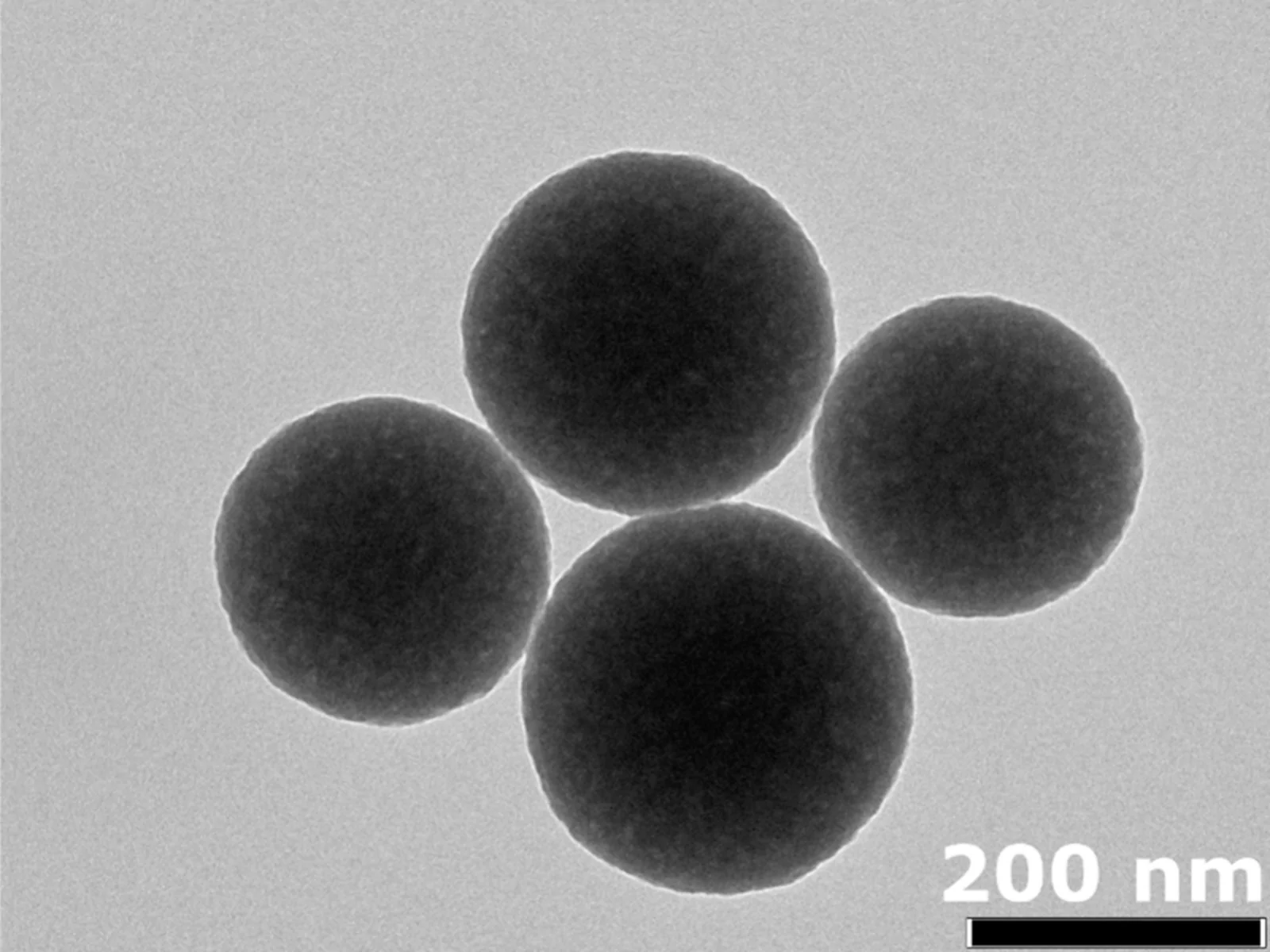
High-resolution transmission electron micrograph of silica synthesized at a stirring rate of 230 mW/kg with porosity indicated by lighter shading within the spheres.

## Conclusion

Mesoporous silica particles of tunable size were synthesized via a modified Stöber method, fully omitting the organic solvent. A surfactant-free emulsion of tetraethyl orthosilicate, ammonia, and water was used in a temperature-controlled batch reactor. Variation of the synthesis temperature from 10 to 30 °C revealed that monodispersity is maintained only above 25 °C; below this threshold, aggregation occurred, which is attributed to decreased particle interaction potentials at lower temperatures. The induction period appears to be critical to determining the final particle size, irrespective of subsequent temperature variations. When the reactor temperature was not kept constant and increased above 25 °C, particles grew to larger sizes than under isothermal conditions, contrary to the expected inverse relationship of the particle size and temperature. The effect is likely explained by the smaller emulsion droplet size obtained, but this phenomenon still requires further experimental confirmation.

The TEOS droplet size was controlled by varying the specific stirring energy from 8 to 1050 mW/kg, producing droplets in the size range of 50–800 μm. Higher stirring energies yielded smaller droplets and, consequently, faster overall reaction rates, with the final silica particle size being inversely proportional to droplet size. Mesoporous silica particles that were 70–350 nm in diameter with pore diameters of 6.6 ± 1.8 nm were obtained at a solid content of up to 9 wt % in a single growth step. A scaling law for the dependence on stirring energy was obtained, which however is only valid at this specific reagent concentration and reactor geometry and is not meant to be general in scope.

Particle growth and completion of the reaction were monitored by photon density wave spectroscopy. The end of the reaction monitored by this method coincided with the cessation of heat evolution, which proceeded considerably faster than in traditional Stöber syntheses. Polydispersities below 2.5% were maintained across all stirring conditions, although particle sphericity is lower for syntheses with low stirring energies. Notably, minimal aggregation was observed in cases where stirring energies were low. Further experiments with a high-shear mixer, at similar stirring energies, produced much larger particles, which were prone to aggregation and secondary nucleation. At similar stirring energies, such mixers provide significantly higher shear rates than conventionally stirred reactors.^48^

The presented method offers a significantly higher space time yield than conventional Stöber syntheses. A major advantage over previous studies is the control of particle size not by variation in reagent concentrations, but rather by direct changes in stirring energy, which affects the droplet size and in turn the growth rates of particles. In future work, a unified model must be introduced, which combines droplet breakage and coalescence,^50^ reactions at the droplet interface,^20^ and aggregative growth of silica particles.^6^ This complex system requires further research. Uncertainty in the droplet size distribution in the reactor leads to uncertainty in the hydrolysis rate. The presence of particles and partially condensed gel particles at the interface is thought to occur, but currently not well characterized, nor is it known if the particles preferably grow at the interface of TEOS and the aqueous phase or if they grow mostly inside the aqueous phase. Viscosity changes in the reactor along the reaction pathway and particle stability due to changes in temperature are further unknown parameters. The current study provides an initial characterization of these phenomena and, we hope, will serve as a foundation for further mechanistic studies.

## Experimental Section

### Synthesis of Silica Particles by Omission of an Organic Solvent

First, 181.82 g (0.873 mol) of tetraethyl orthosilicate (TEOS, >99%, Merck KGaA, Germany) was used as supplied, added to a round-bottom flask with a magnetic stirrer, and equilibrated to the specified reaction temperature. Both a 25 wt % aqueous ammonia solution (Ph. Eur., Carl Roth, Germany, titrated before use) and ultrapure water (Milli-Q-Integral, Merck KGaA) were used as supplied and combined for a total water mass of 326.97 g (18.15 mol) and 90.91 g (5.34 mol) of NH_3_ in a thermostat reactor (Optimax 1001, Mettler Toledo, USA) fitted with a 1 L cylindrical glass vessel with a round bottom (product no. 30230585, Mettler Toledo, USA). The aqueous ammonia solution was then equilibrated in the reactor at the reaction temperature under slow stirring. After temperature equilibration of both reagents, the stirring rate is set to a specified value, and the TEOS is added rapidly. The reactions were terminated after at most 3 h and carried out in duplicate. A complete list of experiments is available in Table S1.

### Reactor Configuration

A milled PTFE lid held the various instruments at fixed heights in the reactor. An overhead stirrer with a stainless steel marine propeller with three blades (8 cm diameter, square pitch) was connected to and suspended 10 mm above the bottom of the reactor vessel. All other instruments, including a digital thermometer, PDW probe, and process microscope (Easyviewer 100, Mettler Toledo, USA), were inserted to a height of approximately 25 cm. The temperature was 55 mm above the bottom of the reactor. The insets act as baffles, enhancing turbulence during mixing. The reactor geometry and its influence on TEOS droplet size were not explored but are important parameters for generating turbulent flow and mediating the droplet size distribution.^34^

Closed loop PID (proportional-integral-derivative) cooling parameters of the reactor were chosen in the iControl software (Mettler Toledo, USA) such that the measured temperature deviated by no more than 2 K of the set temperature at all times. For an additional experiment, the reactor temperature was not PID controlled in order to measure the reaction enthalpy and effects of an increase in temperature (Table S1, entry 12). Thermodynamic effects were estimated via the difference between the reactor temperature (*T*_r_) and the reactor’s jacket temperature (*T*_j_), or Δ*T*. Further details pertaining to the reactor and stirrer geometry may be found in Supplementary Note S2.

High-shear experiments were carried out utilizing a Silverson L5M-A instrument (Silverson Machines Ltd., UK) stirred at 1000 rpm fitted with an emulsion screen in a 400 mL low-form beaker.

### Dynamic and Static Light Scattering

Dynamic light scattering (DLS) and static light scattering (SLS) methods were employed to measure the SP size and distribution (PSD) during synthesis in addition to the data obtained by PDWS and electron microscopy. Samples were taken at specified intervals. DLS measurements were carried out on a Malvern Nano ZS Ultra (Malvern Panalytical, UK). The sample was diluted a hundredfold with absolute ethanol (ROTIPURAN, ≥99.8%, p.a., Carl Roth, Germany). The diluted samples were allowed to equilibrate at 25 °C for at least 60 s.

SLS measurements were carried out with an LS 13 320 laser diffraction particle size analyzer (Beckmann-Coulter, USA). The sample was introduced into the universal liquid module (Beckmann-Coulter, USA) and diluted with ultrapure water. Distribution widths evaluated by SLS were found to deviate from those evaluated by electron microscopy at particle sizes of <200 nm and were not further considered, while the values for mean size correlated well with DLS and electron microscopy.

The refractive index of one sample of particles was measured based on a modified procedure from Nussbaumer et al. with a concentrated aqueous ZnI_2_ solution as the index-matching material.^63^ The dispersion curve deviates minimally from literature for fused silica^64^ (see section S3 for the Cauchy equation describing the experimental refractive index of silica).

### Determination of the TEOS Droplet Size

Images of TEOS droplets and silica agglomerates were recorded during the syntheses with an EasyViewer 100 process microscope (Mettler Toledo, USA). Each image covered an area of 1 mm^2^ in the focal plane. The mean diameter and standard deviation of droplet size are obtained over time by manual evaluation of the droplet sizes in the images. Trends were smoothed by adjacent averaging in a 1 min window.

### Photon Density Wave Spectroscopy

Photon density wave spectroscopy is a multiple-light scattering technique. It allows simultaneous determination of absorption coefficient μ_a_ and reduced scattering coefficient μ_s_^′^ of turbid dispersions. A complete description of the measurement principle and particle size analysis is described by Bressel et al.^21^ A wavelength of 638 nm was selected. Reduced scattering coefficient μ_s_^′^ may be interpreted as the inverse transport mean free path of light where scattering is isotropic. A higher value indicates stronger scattering. Among other factors, μ_s_^′^ is dependent on particle diameter *d*, volume fraction ϕ, and refractive indices *n* of the medium and particles.

### Analysis of the Product Dispersion

The dispersion obtained was centrifuged at a relative centrifugal force of 10000*g* until the supernatant was transparent, which was discarded and replaced with ultrapure water to the same mass, and the particles were redispersed with an immersion ultrasonic disperser (Branson SFX550, Emerson, USA). The centrifugation and washing steps were carried out twice. The solid content was determined in triplicate by gravimetric determination through drying for at least 48 h at 110 °C. Particle yields after washing were typically 90–95% (see Table S1), but this is somewhat variable due to incomplete redispersion of sedimented aggregates; taking representative samples proved to be challenging.

Scanning electron microscopy (SEM) images were recorded on a ZEISS Ultra Plus SEM instrument (Carl Zeiss AG, Germany) at an accelerating voltage of 5 kV. The washed dispersion was diluted a hundredfold with absolute ethanol, and then 10 μL of this dispersion was added to a copper grid with a Formvar film, which was then glued to a sample stub with a carbon glue pad. At least 500 particles were evaluated for each sample from several micrographs utilizing the Hough circle transform algorithm. The PDI of the PSD was evaluated by the square coefficient of variation. Elemental analysis was carried out for C, H, and N on a Vario Micro Cube (Elementar Analysensysteme GmbH, Germany) and for Si on an ICP-OES Spectro Arcos (Spectro Analytical Instruments GmbH, Germany).

For BET analysis, two samples were measured. The first, 215 mg of sample, was dried at 40 °C under vacuum for 72 h and then dried at 200 °C under high vacuum for 3.5 h. The second, 80 mg, was first dialyzed until a constant resistivity of the water was achieved and then freeze-dried for 48 h. The pore size distribution was determined by BJH analysis in the desorption branch. Unfortunately, we were mostly unable to reproduce literature values, which we attribute to pore closing due to sample preparation. The pores are most visible in HRTEM images.

## Supplementary Material



## Notes

Overview of the syntheses carried out (stirring rate, reaction temperatures, reactor type, and yield) (Table S1), extended description of reactor geometry (section S2), Cauchy equation for the refractive index of silica particles (section S3), process microscope images showing droplets of TEOS during the reaction and selected SEM images of particles (section S4), comparison of the reduced scattering coefficient of the herein presented nonethanolic synthesis versus regular Stöber synthesis (section S5), and BJH plot from nitrogen adsorption isotherms compared to pore size obtained by HRTEM (section S6), nitrogen adsorption isotherms (section S7) (PDF)
