# Supplementary material for: Droplet-Mediated Size Control of Stöber Silica Particles in Surfactant- and Organic Solvent-Free Emulsions
Source: Langmuir. 2026 Oct 28;42(44):33060–71. doi: 10.1021/acs.langmuir.6c05490 (PMC13757937; doi:10.1021/acs.langmuir.6c05490)
Supplement: Supplementary file 1 [file la6c05490_si_001.pdf]

Electronic Supplementary Information for "Droplet-Mediated Size Control of Stöber Silica Particles in Surfactant- and Organic Solvent-free Emulsions"

Aaron Justin Koenig*, Jon Scouten, Marvin Münzberg, Ilko Bald*

*Corresponding Author

Aaron Justin Koenig, University of Potsdam, Institute of Chemistry, DE14476 Potsdam, Germany

aaron.koenig@uni-potsdam.de, Orcid: https://orcid.org/0000-0003-3781-5759

Jon Scouten, University of Potsdam, Institute of Chemistry, DE14476 Potsdam, Germany

jon.scouten@uni-potsdam.de, Orcid: https://orcid.org/0009-0002-9852-7208

Marvin Münzberg, University of Potsdam, Institute of Chemistry, DE14476 Potsdam, Germany

mmuenzbe@uni-potsdam.de. Orcid: https://orcid.org/0009-0009-0554-3411

Ilko Bald, University of Potsdam, Institute of Chemistry, DE14476 Potsdam, Germany

ilko.bald@uni-potsdam.de, Orcid: https://orcid.org/0000-0002-6683-5065

Number of pages: 6

Number of figures: 6

Number of schemes: 0

Number of tables: 1

Table of contents

S1: Overview of experiments S2

S2: Description of the reactor geometry S3

S3: Cauchy equation for the refractive index of particles....................S3

S4: SEM and process microscopy images S4

S5: Comparison of $\mu s'$ in aq. synthesis against regular Stöber synthesis S5

S6 Nitrogen adsorption S6

S1: Overview of experiments

*Table S1: Overview of experiments and their experimental parameters. [a] experiments carried out at least twice [b] regular Stöber synthesis *aggregated or sedimented sample*

Experiment Number	*Stirring speed (RPM)*	*Reaction Temperature (°C)*	*Reactor type*	*Yield*	*Notes*
*1[a]**	100	25	OM	114%, 110%	
2[a]	200	25	OM	98.9%, 94%	
3[a]	250	25	OM	89.9%, 94.3%	
4[a]	300	25	OM	95.7%, 96.5%, 93,4%	
5[a]	350	25	OM	93.2%, 95.9%	
6[a]	400	25	OM	90.7%, 96.3%	
7[a]	500	25	OM	95.6%, 95.8%	
*8**	1000	RT	Silverson L5M-A	111.6%	No active temperature compensation
*9**	400	15	OM	Not measured	
*10**	400	20	OM	76.3%	
11	400	30	OM	94.1%	
12[a]	400	25	OM	95.1%, 98.1%	No active temperature compensation
13[a, b]	150	25	OM	98.3%,	Regular Stöber silica for elemental analysis

All reactions were run with the same stoichiometric ratios and amounts as those in the present work, except entry 13 of table S1. This synthesis was carried out with 649.7 mL EtOH, 50 mL TEOS and 100 mL 25% wt% ammonia at 25 °C with stirring at 150 rpm. TEOS was prediluted with 100 g of the ethanol, then added to the rest of the ethanol and ammonia in the already stirring reactor.

S2: Description of the reactor geometry

All reactions were carried out on an OptiMax 1001 synthesis platform fitted with a 1L borosilicate glass reactor vessel (Art. No. 30230585, Mettler Toledo, USA), inner Ø 101 mm, height 172 mm, 3 mm wall thickness. A PTFE reactor cover (Part No. 51162634, Mettler Toledo, USA) held the instruments in place. A Pt100 temperature sensor (Part No. 30090590, Mettler Toledo, USA) with an outer Ø of 3.2 mm, and the EasyViewer 100 with an outer Ø of 9.5 mm (Part No. 14000210, Mettler Toledo, USA) were inserted to a height of 55 mm. A PDW probe was inserted to a height of 55 mm. It consists of a 25 mm Ø stainless steel tube, at the end of which are 8 stainless steel cannulas (1.6 mm Ø, 70 mm length) inside of which are flat polished bare fiber optics. The stirrer shaft with an outer Ø of 10 mm is fitted with a marine propeller 3-blade type stirrer (70 mm diameter, 28 mm individual blade length, 45° blade angle).

S3: Cauchy equation for the refractive index of particles

Real refractive indices n_{real} of particles synthesized by the aqueous Stöber method were measured as described in the paper. A concentrated aqueous ZnI_2 solution with known refractive index was used. Its refractive index was measured on a DSR-λ refractometer (SCHMIDT + HAENSCH GmbH & Co., Germany). The resulting Cauchy-Equation for the Stöber particles is given here with deviations. The wavelength λ is given in units of nm.

$$n_{real} = (1.44407 \pm 0.0011) + \left(\frac{6799 \pm 741}{\lambda^2}\right) + \left(\frac{-3.14 \cdot 10^8 \pm 1 \cdot 10^8}{\lambda^4}\right)$$

S4: SEM and process microscopy images

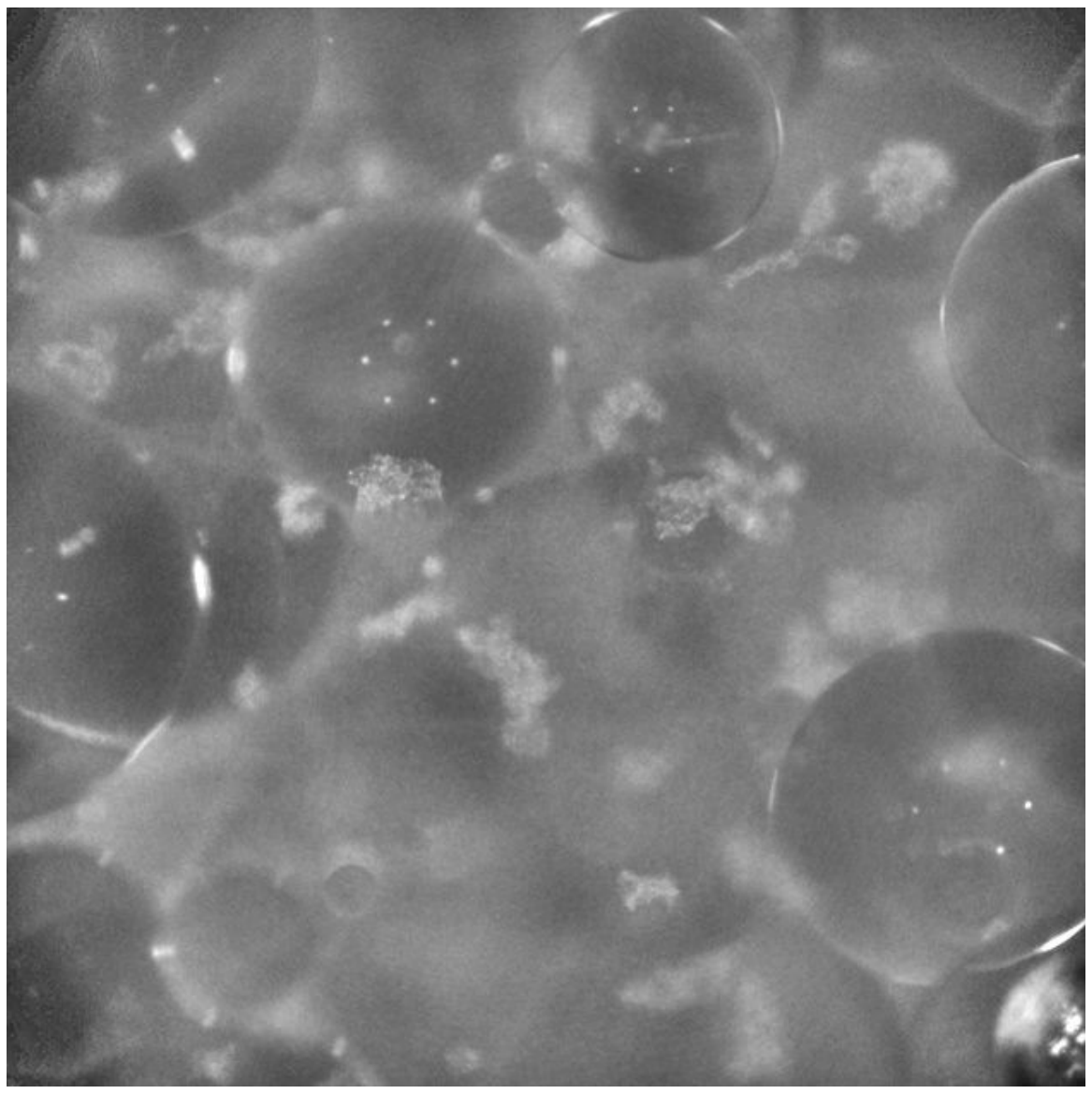

Figure S4a: Process microscopy image of synthesis 6 in table S1 at a reaction time of 2 minutes and 6 seconds after addition of TEOS. Both TEOS droplets and large aggregates of silica are visible. The aggregates make up less than 4% of the total volume of all particles in dispersion after completion of the synthesis.

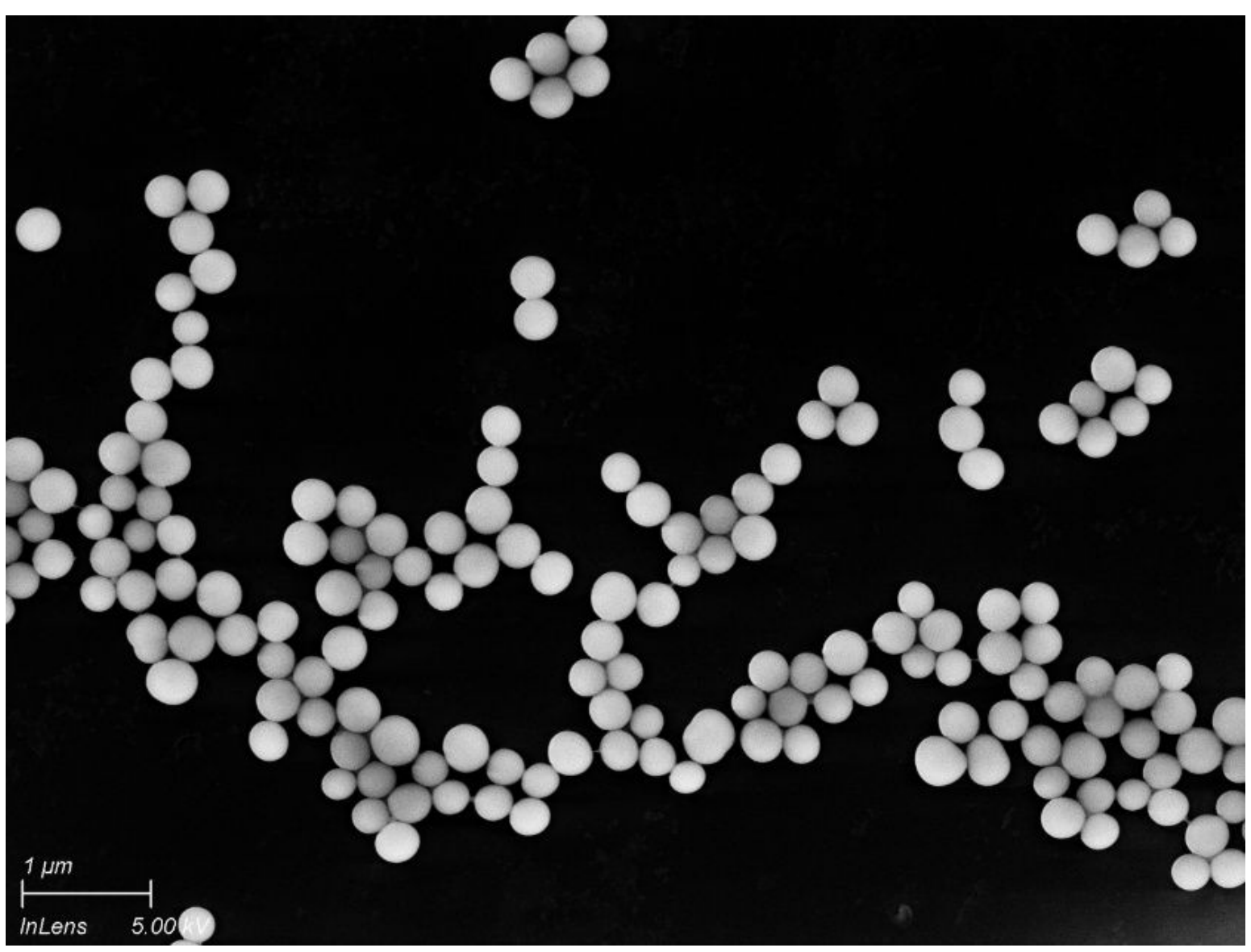

Figure S4b: SEM image of particles from synthesis with entry 6 in table S1.

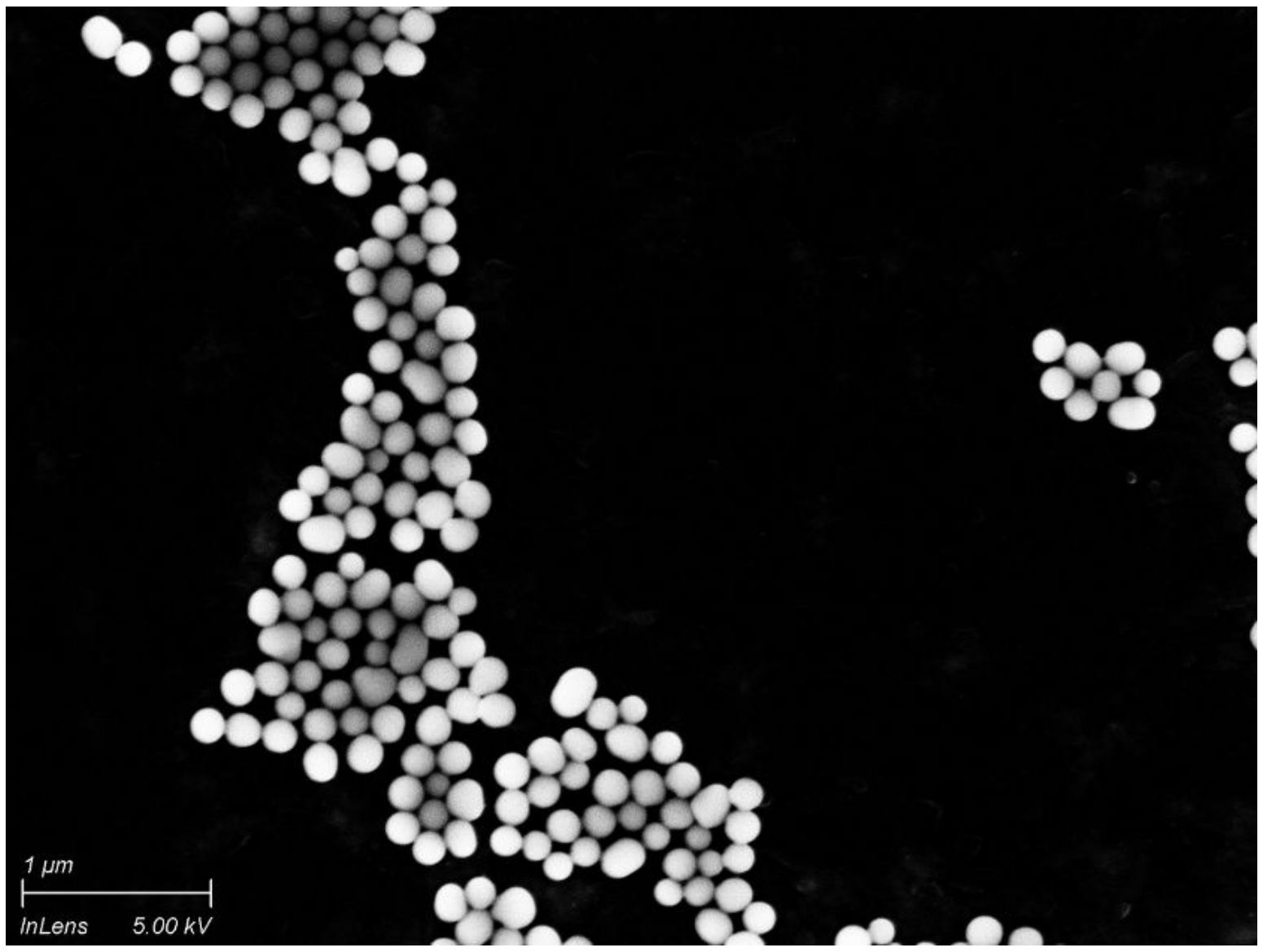

Figure S4c: SEM image of particles from synthesis with entry 12 in table S1

S5: Comparison of μ_s' in aq. synthesis against regular Stöber synthesis

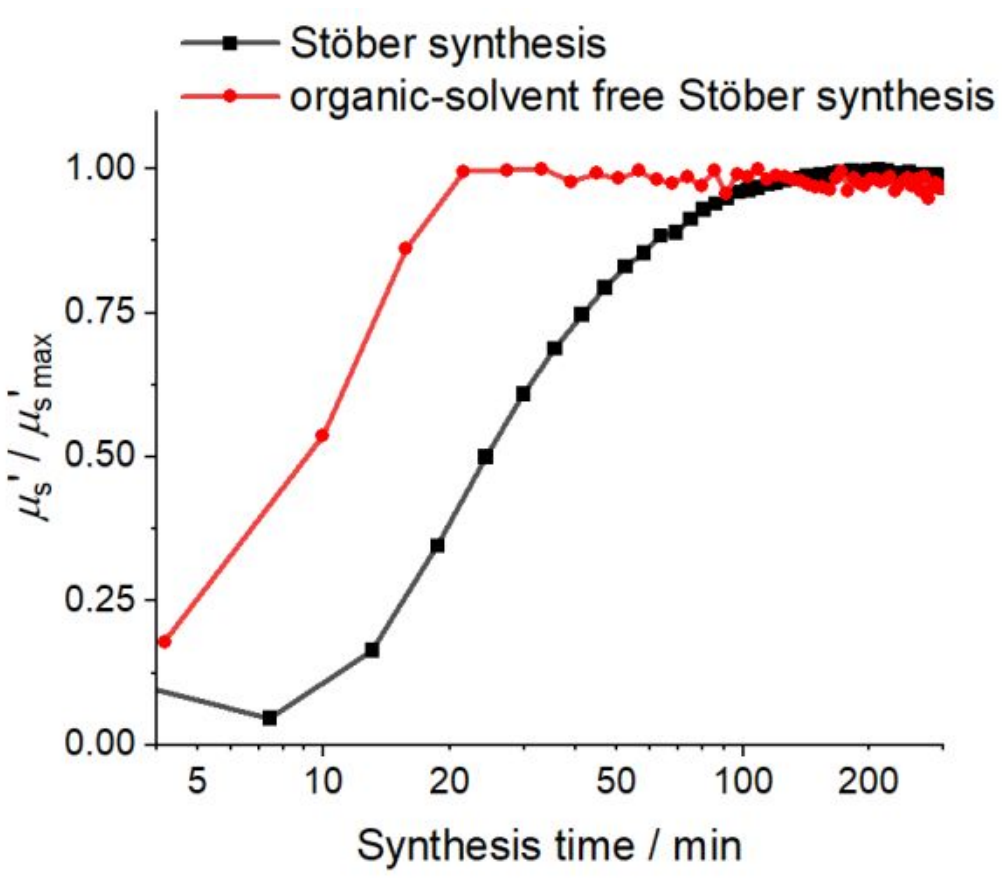

Figure S5: Normalized reduced scattering coefficient trends at a wavelength of 638 nm from synthesis 4 from table S1 (red) and a regular Stöber synthesis, synthesis 13 from table S1 (black).

S6 Nitrogen adsorption

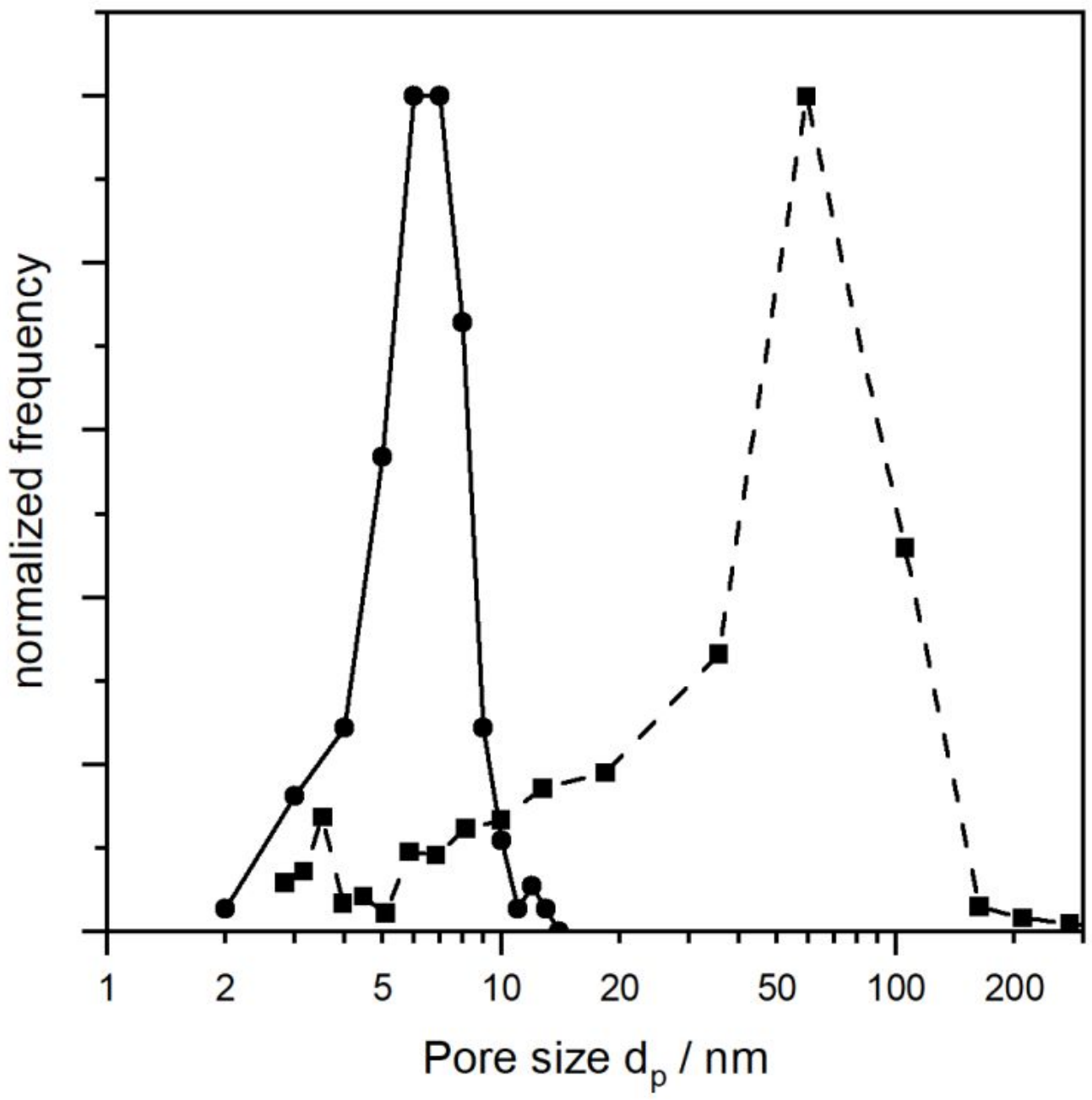

Figure S6: Comparison between porosity obtained by BET / BJH experiments (dashed line) and by manual evaluation of high-resolution TEM (solid line). The distribution generated by BJH likely accounts for interparticle pores, not intraparticle pores. Experiment 5 in Table S1 was used for evaluation.

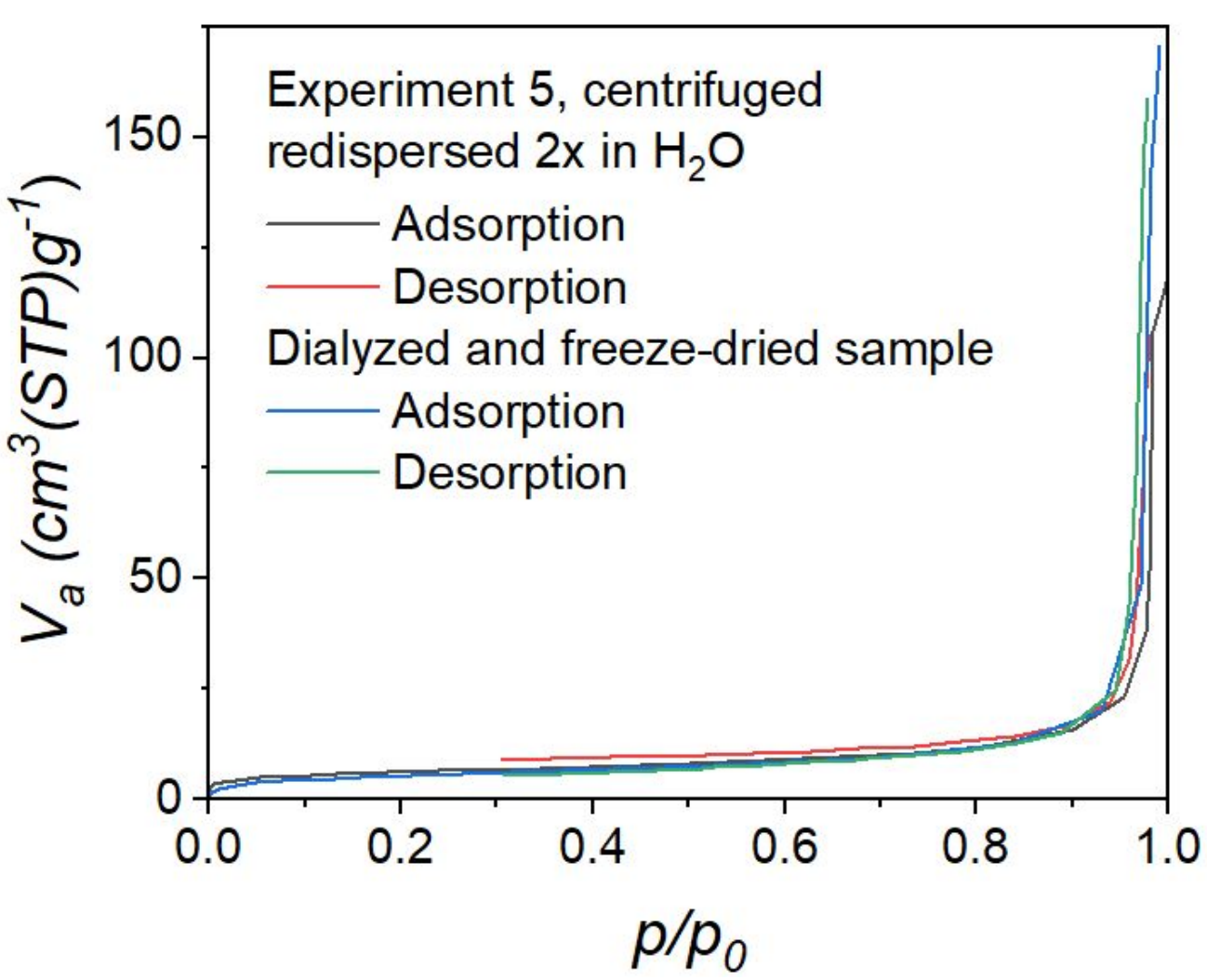

Figure S7: Nitrogen adsorption isotherm for two samples from experiment 5, prepared in different ways. Due to the type II isotherm, the results point to a non-porous material with only interparticle pores, contrary to what is observed with HRTEM, where the pores are clearly visible.
